# Comparison of Humoral Immune Responses to Different Forms of *Salmonella enterica* Serovar Gallinarum Biovar Gallinarum

**DOI:** 10.3389/fvets.2020.598610

**Published:** 2020-11-06

**Authors:** Nam-Hyung Kim, Eun-Jin Ha, Dae-Sung Ko, Kang-Seuk Choi, Hyuk-Joon Kwon

**Affiliations:** ^1^Laboratory of Poultry Medicine, Seoul National University, Seoul, South Korea; ^2^Research Institute for Veterinary Science, College of Veterinary Medicine, BK21 for Veterinary Science, Seoul, South Korea; ^3^Laboratory of Avian Diseases, College of Veterinary Medicine, Seoul National University, Seoul, South Korea; ^4^Farm Animal Clinical Training and Research Center (FACTRC), GBST, Seoul National University, Seoul, South Korea

**Keywords:** *Salmonella serovar* Gallinarum biovar Gallinarum, humoral immunity, vaccines, natural infection, Peptide-ELISA

## Abstract

Fowl typhoid is caused by *Salmonella enterica* serovar Gallinarum biovar Gallinarum (SG), and live attenuated, rough vaccine strains have been used. Both humoral and cellular immune responses are involved in protection, but the humoral responses to different forms of SG antigens are unclear. In this study, we compared humoral responses to a killed oil-emulsion (OE) smooth vaccine (SG002) and its rough mutant vaccine (SR2-N6) strains using proteomics techniques. We identified two immunogenic outer membrane proteins (OmpA and OmpX), and the selected linear epitopes were successfully applied in peptide-ELISA. Our peptide- and total OMP-ELISAs were used to compare the temporal humoral responses to various SG antigens: OE SG002 and SR2-N6; live, killed [PBS-suspension (PS) and OE)] and mixed (live and PS) formulations of another rough vaccine strain (SG 9R); and orally challenge with a field strain. Serum antibodies to the linear epitopes of OmpA and OmpX lasted only for the first 2 weeks, but serum antibodies against OMPs increased over time. The rough strain (SR2-N6) and mixed SG 9R induced higher serum antibody titers than the smooth strain (SG002) and single SG 9R (OE, live and PS SG 9R), respectively. Infection with the field strain delayed the serum antibody response by ~2 weeks. Mucosal immunity was not induced by any formulation, except for infection with the field strain after SG 9R vaccination. Thus, our results may be useful to understand humoral immunity against various SG antigens and to improve vaccine programs and serological diagnosis in the field.

## Introduction

*Salmonella enterica* serovar Gallinarum biovar Gallinarum (SG) is a pathogen causing fatal and persistent infection, fowl typhoid (FT) (1, 2). Both humoral and cell-mediated immune responses are required to prevent mortality and achieve bacterial clearance (3). A live vaccine strain, SG 9R, mimics infection of pathogenic field strains, and has been used to prevent FT worldwide (4).

The potent immunostimulatory effect of lipopolysaccharide (LPS) is mediated by O-Ag and lipid A, which induce T cell-independent humoral and TLR4-mediated innate immune responses, respectively (5). Although LPS induces a strong humoral immune response to concomitantly inoculated antigens, LPS on the surface of bacteria may also shield or compete with outer membrane proteins (OMPs), resulting in decreased immunogenicity of OMP (6, 7). Therefore, while SG 9R is a rough strain with defective outer-core and O-antigen regions (O-Ag) of lipopolysaccharide (LPS), it may induce a different humoral immune response from field strains against OMP (8). The protective efficacy of OMP vaccines has already been established, and protective OMPs of *S.enterica* serovars have been identified for vaccine development (9, 10).

Although SG 9R has been commonly used in the field, it displays potential pathogenicity and may cause mortality and gross lesions in the liver under immunosuppressive conditions (8). Therefore, SG 9R was not recommended for use in chicks under 6 weeks old (w-o) who are most susceptible and may become carriers (4, 11). For this reason, killed vaccines, if possible, need to be considered, but basic data on the differences in humoral immune responses to different forms of SG antigens (oil-emulsion, killed, smooth vs. rough SG; live vs. killed with or without oil adjuvant vs. a mixture of live and killed SG 9R; or field strain) are insufficient. In addition, humoral immunity against natural infection with field strains is unclear. Humoral immunity to live or killed bacteria is the sum of antibodies directed to multiple antigens and their epitopes. Therefore, investigations of a single epitope-specific antibody in the antiserum against different antigens using single peptide epitopes may provide more insights into the kinetics of humoral immunity. In this study, we compared humoral immune responses to smooth and rough SG strains and identified immunogenic OMPs and their linear epitopes. We developed linear epitope-based peptide-ELISAs to compare humoral immune responses to different forms of SG antigens, and the results were compared with data from the OMP-ELISA.

## Materials and Methods

### Bacteria, Serum Samples, and Experimental Birds

A commercial rough vaccine strain, SR2-N6 (DAE SUNG Microbiological Lab., Uiwang-si, Korea), and its parent strain SG002 were used to compare the effect of LPS on humoral immunity, and a commercial rough vaccine strain, SG 9R, was purchased from the manufacturer (Nobilis; Intervet International, Boxmeer, the Netherlands) (12). SG0197, a virulent strain isolated from commercial chickens in 2001, was used to observe the immune response of challenged chickens (12). The strains were cultured in Luria-Bertani broth (Duchefa Biochemie, Groot Bijgaarden, Belgium) with shaking at 37°C overnight.

One d-o male Hy-Line brown layer chicks without SG vaccination were purchased from a farm (Yangji Farm, Pyeongtaek-si, Korea) and reared for animal experiments to compare humoral immune responses to different forms of SG antigens. Feed and water were supplied *ad libitum*.

Fifty-six field serum samples obtained for serological tests from 6 layer and breeder farms were used to determine humoral immunity to SG in the field. In detail, L1D included 10 samples from 1-day-old (d-o) layer chicks, L12W included 10 samples from 12-w-o layer chickens vaccinated at 10 w-o, L19W included 10 samples from 19-w-o layer chickens vaccinated twice at 8 and 15 w-o, and L41W included 10 samples from 41-w-o layer chickens vaccinated twice at 7 and 16 w-o. PS18W and PS23W included 10 and 6 samples from 18 and 23-w-o parent stocks, respectively.

### 2D-Gel Electrophoresis, Immunoblotting, and LC-MS/MS

Total bacterial proteins were extracted via cell lysis with 7 M urea, 2 M thiourea, 4% CHAPS (3-[(3-cholamidopropyl)dimethylammonio]-1-propanesulfonate), and 2.5% dithiothreitol (DTT) and quantified with the Bradford protein assay. OMPs were extracted using the previously described sodium lauroyl sarcosine (SLS) method (13), with some modifications. Briefly, the cultured bacteria were centrifuged and the pellet was washed with 50 mM Tris-HCl. After centrifugation, lysis buffer (50 mM Tris-HCl and 150 mM NaCl) was added, and the cells were lysed by ultrasonication. The supernatant was ultracentrifuged at 100,000 × g for 1 h. Pellets were resuspended in 2% SLS and 50 mM Tris-HCl, and then incubated at room temperature for 40 min. After another ultracentrifugation step, pellets were stored by adding 1% Triton X-100 to the lysis buffer.

2D-gel electrophoresis of total proteins or OMPs was performed using isoelectric focusing (pH 3-10 for whole bacteria or pH 4-7 for OMPs) and 14% SDS-PAGE gels, and separated proteins were electrotransferred to nitrocellulose membranes for western blotting (ProteomeTech, Seoul, Korea) (14). Membranes were incubated with anti-SR-N6 (1:10,000 dilution) and anti-SG002 (1:5,000 dilution) serum samples. LC-MS/MS was performed as described below. The analysis was performed using a nano ACQUITY UPLC and LTQ-Orbitrap-mass spectrometer (Thermo Electron, San Jose, CA). One of the mobile phases for LC separation was 0.1% formic acid in deionized water, and the other was 0.1% formic acid in acetonitrile. The flow rate was 0.5 μl/min, and the transfer tube temperature was set to 160°C. The MS/MS data were interpreted using SEQUEST software (Thermo Quest, San Jose, CA, USA), and the generated peak lists were compared using the MASCOT program (Matrix Science Ltd., London, UK).

### B Cell Epitope Prediction and Peptide Synthesis

B cell epitopes were predicted by the IEDB B cell epitope prediction program (http://tools.iedb.org/bcell/), and they were located on the 3D structure files of corresponding proteins generated with PyMOL 2.2 (Schrodinger, New York, USA). Selected peptides were synthesized with a modification of the N-terminus by adding aminocaproic acid for better performance of the peptide-ELISA (Cosmogenetech, Seoul, Korea).

### ELISA

Synthesized peptides (1 μl/ml) or SG 9R OMP extracts (105 ng/ml) in 100 mM sodium bicarbonate/carbonate coating buffer (pH 9.6) were used to coat an immunoplate (SPL Life Science, Pocheon-si, Korea) at 4°C overnight. Antigen-coated wells were washed twice with PBST (PBS containing 0.5% Tween 20) and blocked with 1% bovine serum albumin (BSA) (GenDEPOT, Katy, USA) at room temperature for 2 h. After washing the plates as described above, the primary antibody, which was serum or bile juice (1:300 in PBST containing 1% BSA), was added, incubated for 30 min, and then the plate was washed 4 times with PBST. The secondary antibody, an HRP-conjugated goat-anti chicken IgG or IgA antibody (Bethyl, Laboratories, Montgomery, USA; diluted 1:10,000 in PBST containing 1% BSA), was added for 30 min, and the plate was washed as described above. TMB substrate (SurModics, Eden Prairie, USA) was added for 10 min, and the OD was measured at 450 nm after the addition of stop solution. We used a commercial *Salmonella* D group ELISA kit to test the anti-O-Ag antibody according to the manufacturer's recommendation (BioChek BV., Reeuwijk, the Netherlands).

### Inactivation and Preparation of Oil-Emulsion (OE) SG

Cultured bacteria were centrifuged and washed once with PBS. Bacteria were inactivated at 65°C for 2 h in a water bath and cooled gradually to room temperature. The inactivation was confirmed by culture on Mueller Hinton Agar (Duchefa Biochemie, Groot Bijgaarden, Belgium). The live and heat-inactivated bacteria were diluted to 1 × 10^7^ cfu/100 μl and 1 × 10^9^ cfu/100 μl in PBS, respectively. The live and killed mixture was prepared by mixing the same volume of both preparations of bacteria to obtain 200 μl. The OE bacteria were prepared by emulsifying heat-inactivated bacteria with oil adjuvant (Montanide ISA 70, Seppic Co., Courbevoie, France) at a ratio of 3 to 7 (~3.3 × 10^8^ cfu/100 μl of OE) (Table 1).

**Table 1:** Inoculated vaccines and field strain.

**Sample**	**Solvent**	**Dose (cfu/chicken)**	**Inoculation route**
SR2-N6	PBS with the ISA 70 adjuvant	3 × 10^8^	IM
SG002	PBS with the ISA 70 adjuvant	3 × 10^8^	IM
SG9R	PBS	1 × 10^7^	IM
OE SG9R	PBS with the ISA 70 adjuvant	3 × 10^8^	IM
PS SG9R	PBS	1 × 10^9^	IM
MX SG9R	PBS	1 × 10^7^(SG9R)+1 × 10^9^(PS SG9R)	IM
SG197	PBS	1 × 10^6^	*per os*

### Animal Experiments

Fifteen (5 chickens in each group) 3-w-o male brown layer chickens were divided into SR2-N6, SG002, and negative control groups to compare the humoral immune responses to smooth (SG002) and rough (SR2-N6) strains, respectively. OE SR2-N6 and OE SG002 were inoculated via the intramuscular route (100 μl/chicken), and serum samples were collected weekly for up to 3 weeks postinoculation (wpi). Bile juice samples were collected from the gall bladder at 3 wpi with a 1 ml syringe. Specific antibodies in serum (IgG) and bile juice (IgA) samples were measured using the ELISA.

Forty 3-w-o male brown layer chickens were assigned to OE SG 9R (10), live SG 9R (10), mixed SG 9R (10), PS (PBS –suspension) SG 9R (5), and negative control groups (5) to compare the humoral immune responses to different forms of SG 9R. All groups were inoculated via the intramuscular route (100 μl/chicken), and serum samples were collected weekly for up to 3 wpi. Bile juice samples were collected at 3 wpi as described above. Specific antibodies in serum and bile juice samples were measured using the ELISA.

Fifteen 4-w-o chickens were infected with the field strain (SG0197, 1 × 10^6^ cfu/0.1 ml/chicken) *per os*, and serum samples were collected from the surviving chickens weekly for 4 wpi. Bile juice samples were collected after 3 days of starvation at 4 wpi, as described above. Specific antibodies in serum and bile juice samples were measured using the ELISA.

Twenty 6-w-o chickens were divided into SG 9R vaccine and no vaccine groups to compare the mucosal immune responses. The SG 9R vaccine group was vaccinated with SG 9R (1 × 10^7^ cfu/100 μl/chicken) via the intramuscular route, and both groups were challenged with SG0197 at 2 wpi (8 w-o). After 2 wpi, SG0197 bile juice samples were collected as described above, and specific antibodies were measured using the ELISA.

All animal experiments were approved by the Institutional Animal Care and Use Committee of BioPOA Co. (permission number BP-2019-C31-1).

### Statistical Analysis

Analyses were performed with SPSS Statistics version 26.0 (SPSS, Chicago, IL, USA). One-way ANOVA was used to analyze significant differences between the groups, followed by the Bonferroni *post-hoc* test (Figure 2C 1 wpi and 3 wpi, Figure 2D 1 wpi, Figures 5A,C). When unequal variance was observed, the Welch test was used for the analysis, and the Games-Howel test was performed as the *post hoc* test (Figure 2B 2 wpi, Figure 2C 2 wpi, Figure 2D 2 wpi and 3wpi, Figure 3A 2 wpi, Figure 3B 2 wpi, Figure 3C 1 wpi, Figure 3D 1-3 wpi). Data with a non-normal distribution were subjected to the Kruskal-Wallis *H*-test, and the Bonferroni correction was used as the *post-hoc* test (Figure 2A 1-3 wpi, Figure 2B 1 wpi and 3 wpi, Figure 3A 1 wpi and 3 wpi, Figure 3B 1 wpi and 3 wpi, Figure 3C 2 wpi and 3 wpi, Figures 4, 5B,D, 6). If only two groups were analyzed, the significance was determined with the *t*-test for data with a normal distribution (Figures 5E–G), and the Mann-Whitney *U*-test was used for data with a non-normal distribution (Figures 5H,I). Statistical significance was considered when the *p* < 0.05.

## Results

### Comparison of Humoral Immune Responses to Smooth (SG002) and Rough (SR2-N6) Strains

**Figure 1:**
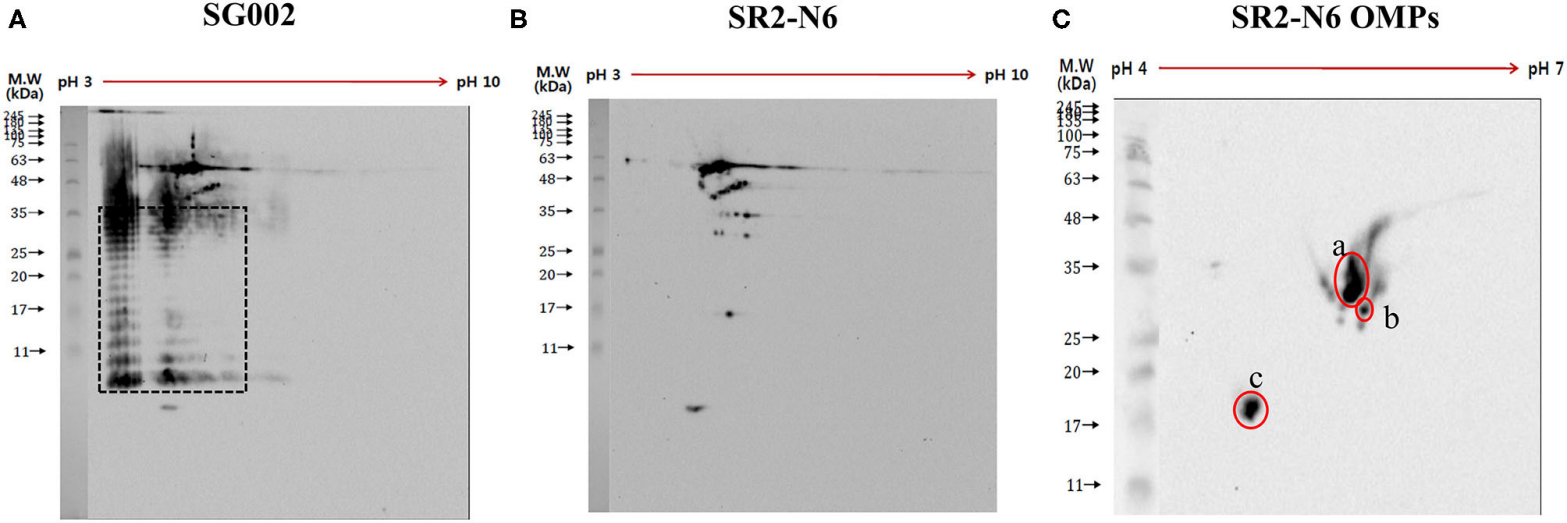
Antigens of SG002 were tested against antiserum of SG002 **(A)** and SR2-N6 **(B)** using 2DE western blotting. Different blots obtained in **(A,B)** were analyzed using LC-MS/MS (Supplementary Table 1). Distinct ladders at different isoelectric points represented the O antigen (black dotted rectangle). Omp extracts of SR2-N6 were also tested against the antiserum of SR2-N6 **(C)** using 2DE western blotting. Three spots (a, b, and c) were analyzed with LC-MS/MS (Table 2).

In contrast to anti-SR2-N6 serum samples, anti-SG002 serum samples showed a strong antibody reaction to O-Ag (at least two distinct ladders at different isoelectric points) (Figure 1A, black dotted rectangle). The different spots recognized by the anti-SR2-N6 serum sample were analyzed with LC-MS/MS. Interestingly, most of the spots were not OMP and included translation elongation factor G, GroEL, phosphoglycerate kinase, elongation factor Tu, electron transfer flavoprotein subunit beta, *etc*. (Supplementary Table 1). To identify immunogenic OMPs, we performed 2D-gel electrophoresis and immunoblotting with OMPs of SR2-N6 and anti-SR2-N6 serum samples to identify major antigens. The three major antigen spots were identified to be OmpA (spots a and b) and OmpX (spot c) (Figure 1C, Table 2). We selected candidate peptides for the peptide-ELISA according to the amino acid sequences of OmpA (CAR36850) and OmpX (CAR36706) (Table 3).

**Table 2:** Proteins predicted by the LC-MS/MS analysis.

**Spot label**	**NCBI BLAST**	**Protein name**	**Score**	**Mass**
**a**	WP_065702086.1	porin OmpA [*Salmonella enterica*]	4039	37640
**b**	WP_065702086.1	porin OmpA [*Salmonella enterica*]	1735	37640
**c**	WP_058343733.1	outer membrane protein OmpX [*Salmonella enterica*]	3409	17570

**Table 3:** B cell epitopes of OmpA and OmpX tested in the peptide-ELISA.

**Protein/location**	**Peptide name**	**Sequence (N- to C-terminus)**
OmpA/N-terminus	OmpA-N-L3	TKSNV PGGPS
	OmpA-N-L4	TNNIG DANTI GTR
OmpA/C-terminus	OmpA-C-L1	QLYSQ LSNLD PKDGS
	OmpA-C-L2	GESNP VTGNT CDNVK
OmpX	OmpX-L2	GKFQT TDYPT YKHDT

**Figure 2:**
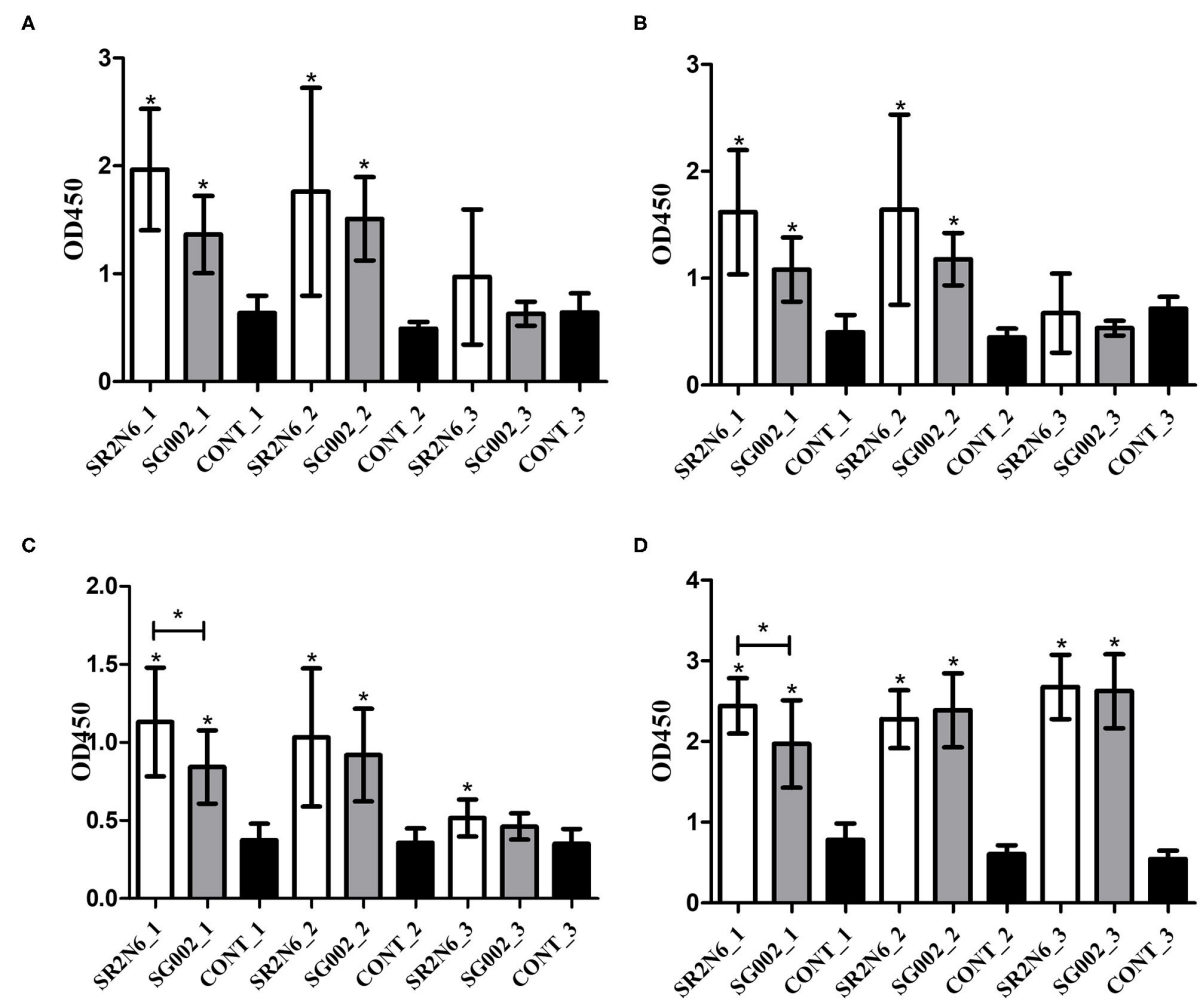
Humoral immune responses to the smooth strain (SG002) and rough strain (SR2-N6) measured using ELISAs (mean with SD): OmpA-N-L3 **(A)**, OmpA-N-L4 **(B)**, OmpX-L2 **(C)**, and OMP ELISAs **(D)**. *Indicates a significant difference [*P*-values - **(A)** SR2-N6 vs. CONT at 1 wpi (0.000) and 2 wpi (0.001); SG002 vs. CONT at 1 wpi (0.008) and 2 wpi (0.000); **(B)** SR2-N6 vs. CONT at 1 wpi (0.000) and 2 wpi (0.005); SG002 vs. CONT at 1 wpi (0.01) and 2 wpi (0.000); **(C)** SR2-N6 vs. SG002 at 1 wpi (0.046); SR2-N6 vs. CONT at 1 wpi (0.000), 2 wpi (0.002), and 3 wpi (0.003); SG002 vs. CONT at 1 wpi (0.001) and 2 wpi (0.000); **(D)** SR2-N6 vs. SG002 at 1 wpi (0.035); SR2-N6 vs. CONT at 1 wpi (0.000), 2 wpi (0.000), and 3 wpi (0.000), SG002 vs. CONT at 1 wpi (0.000), 2 wpi (0.000), and 3 wpi (0.000)].

A pilot study with the synthesized peptides (OmpA-N-L1, OmpA-N-L2, OmpA-N-L3, OmpA-N-L4, OmpX-L1, and OmpX-L2) and anti-SG002 and anti-SR2-N6 serum samples revealed that the reactivity of OmpA-N-L1, OmpA-N-L2, and OmpX-L1 was too low to differentiate responses from anti-SG002 and anti-SR2-N6 serum samples. We selected OmpA-N-L3, OmpA-N-L4, and OmpX-L2 for the peptide-ELISA. According to the results, the anti-SR2-N6 antibody titer was significantly higher than the anti-SG002 antibody titer in the OmpX-L2 and OMP-ELISA at 1 week postinoculation (wpi) (Figures 2C,D). All the anti-SR2-N6 and anti-SG002 serum samples showed significantly higher OD values than the negative control only for the first 2 weeks using peptide-ELISAs, except for OmpX_L2. However, in the OMP-ELISA, significantly higher OD values were observed than the negative control, with a gradual increase during the observation period.

### Comparison of Humoral Immune Responses to Live, Killed and Mixture of Live, and Killed Rough Vaccine Strains (SG 9R)

**Figure 3:**
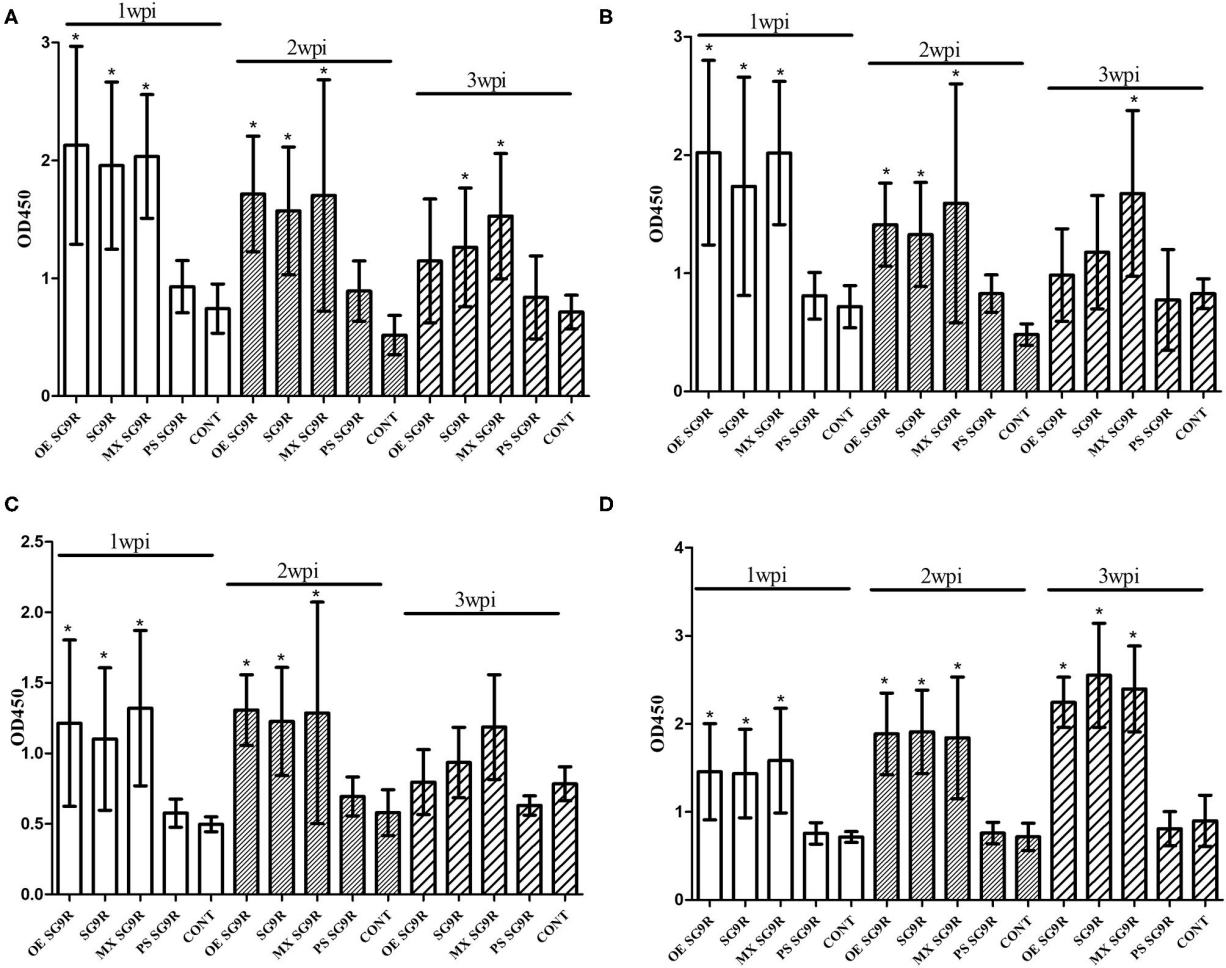
Humoral immune responses measured using ELISAs under various conditions (mean with SD): OmpA-N-L3 **(A)**, OmpA-N-L4 **(B)**, OmpX-L2 **(C)**, and OMP-ELISA **(D)**. SG 9R was inoculated as killed adjuvanted (OE), live, mixed with live and killed (MX), or killed bacteria in a PBS suspension (PS) and compared with the negative control (CONT). *Indicates a significant difference compared with CONT [*P*-values - **(A)** OE SG 9R at 1 wpi (0.000) and 2 wpi (0.000); SG 9R at 1 wpi (0.000), 2 wpi (0.000), and 3 wpi (0.048); MX SG 9R at 1 wpi (0.000), 2 wpi (0.000), and 3 wpi (0.000); **(B)** OE SG 9R at 1 wpi (0.001) and 2 wpi (0.000); SG 9R at 1 wpi (0.019) and 2 wpi (0.000); MX SG 9R at 1 wpi (0.000), 2 wpi (0.001), and 3 wpi (0.022); **(C)** OE SG 9R at 1 wpi (0.000) and 2 wpi (0.000); SG 9R at 1 wpi (0.000) and 2 wpi (0.000); MX SG 9R at 1 wpi (0.000) and 2 wpi (0.006); **(D)** OE SG 9R at 1 wpi (0.013), 2 wpi (0.000), and 3 wpi (0.000); SG 9R at 1 wpi (0.009), 2 wpi (0.000), 3 wpi (0.000); MX SG 9R at 1 wpi (0.008), 2 wpi (0.004), and 3 wpi (0.000)].

The OD values of anti-OE SG 9R, anti-live SG 9R, and anti-mixed SG 9R serum samples were not significantly different from each other, and produced higher OD values than the anti-PS SG 9R serum samples and negative control samples at 1 and 2 wpi in the peptide-ELISAs (Figure 3). Interestingly, anti-mixed SG 9R showed significantly higher OD values than the negative control in the OmpA-N-L3 and OmpA-N-L4 peptide-ELISAs at 3 wpi. According to the results of the OMP-ELISA, anti-OE SG 9R, anti-SG 9R, and anti-mixed SG 9R serum samples showed significantly higher OD values than the negative control samples with a gradual increase over time. Anti-PS SG 9R serum samples did not show significantly higher OD values than the negative control in either peptide- or OMP-ELISAs.

We tested two additional peptides (OmpA-C-L1 and OmpA-C-L2) in the C-terminal domain of OmpA using the peptide-ELISA (Table 3). The anti-OE SG 9R serum samples showed significantly higher OD values than the negative control at 1 and 2 wpi, but not at 3 wpi (Supplementary Figure 1).

### Humoral Immunity Against Natural Infection With a Field Strain (SG0197)

**Figure 4:**
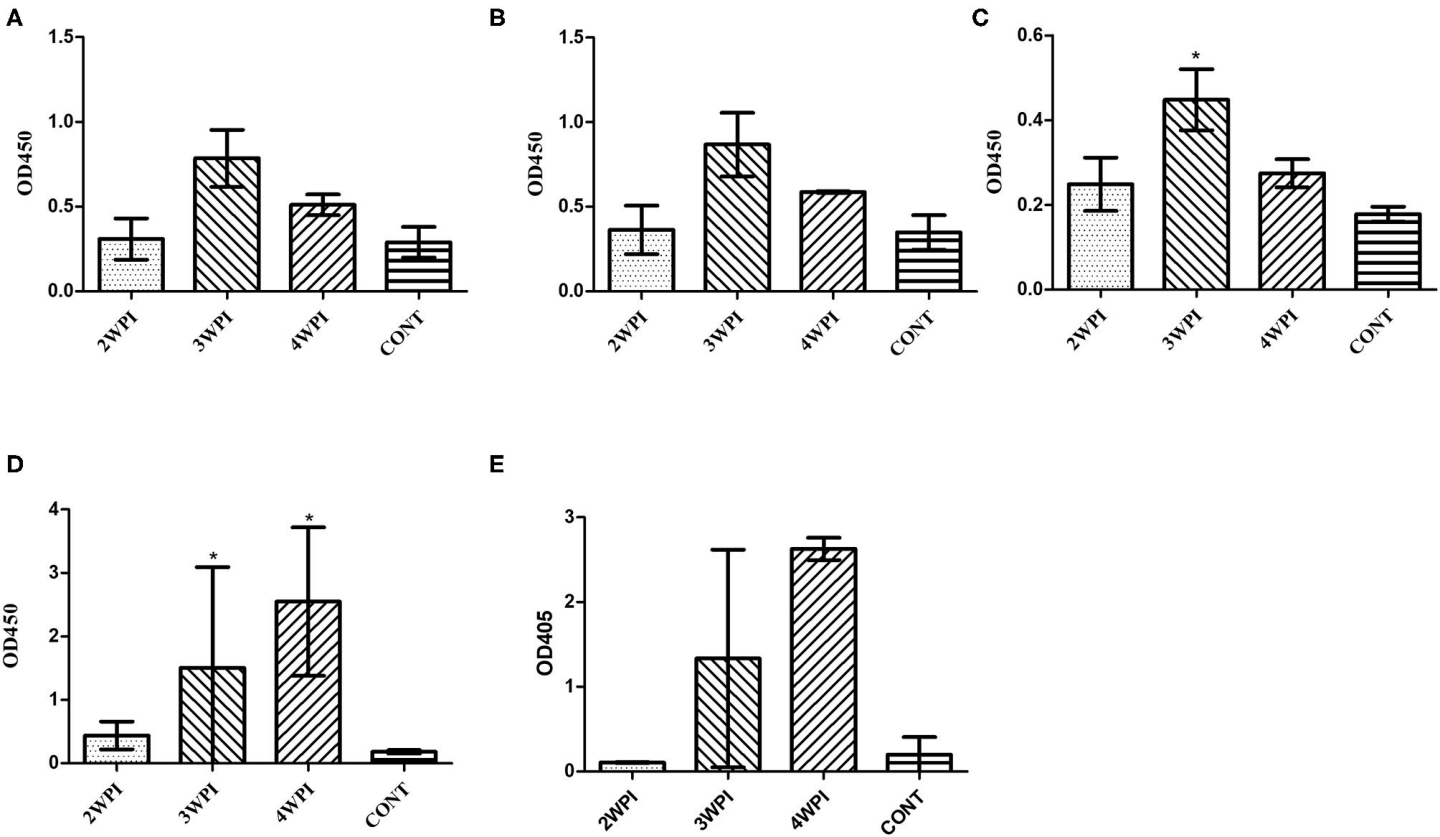
Evaluation of humoral immunity in response to challenge with a field strain (mean with SD) using the OmpA-N-L3 **(A)**, OmpA-N-L4 **(B)**, OmpX-L2 **(C)**, OMP-ELISA **(D)**, and *Salmonella* D group O-Ag ELISA **(E)**. The field strain SG0197 was infected *per os* at 4 weeks of age, and serum samples were collected weekly for 4 weeks. *Indicates a significant difference compared with CONT [*P*-values - **(C)** 3 wpi (0.016); **(D)** 3 wpi (0.036) and 4 wpi (0.017)].

SG0197 infection caused 86.7% (13/15) mortality within 4 weeks (7/15 at 2 wpi, 5/15 at 3 wpi and 1/15 at 4 wpi); therefore, the numbers of serum samples were 8 at 2 wpi, 3 at 3 wpi, and 2 at 4 wpi as the number of surviving chickens decreased. The serum samples from the challenged group only showed significantly higher OD values than the negative control group at 3 wpi using peptide-ELISAs (Figure 4C) but significantly higher OD values at 3 and 4 wpi than the negative control group using the OMP-ELISAs (Figure 4D).

### Comparison of Mucosal Immunity Against Various SG Antigens

**Figure 5:**
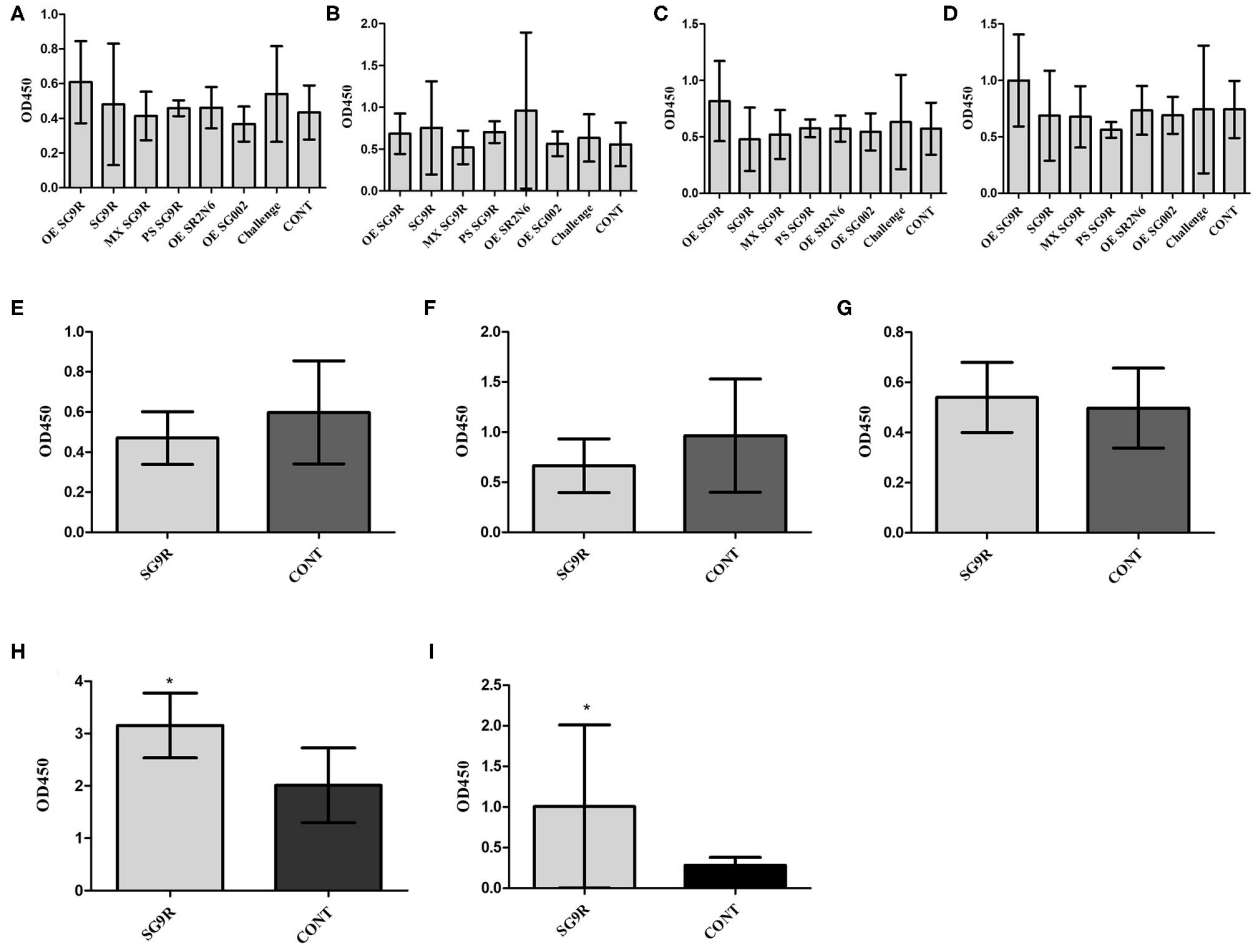
The mucosal immunity of bile was measured with IgA secondary antibody with the same ELISA (mean with SD): OmpA-N-L3 **(A,E)**, OmpA-N-L4 **(B,F)**, OmpX-L2 **(C,G)**, OMPs **(D,H)**, and O-Ag IgA ELISA **(I)**. Birds were inoculated with the vaccine at 3 weeks of age, and bile juice was collected at 3 wpi **(A–D)**. Eight-week-old brown layer chickens with or without SG 9R vaccination were challenged with SG0197 at 6 weeks of age, and bile juice samples were collected at 10 weeks of age **(E–I)**. *Indicates a significant difference compared with CONT [*P*-values - **(H)** (0.001) and **(I)** (0.001)].

The anti-OE SG 9R, anti-SG 9R, anti-mixed SG 9R, anti-PS SG 9R, anti-OE SR2-N6, and anti-OE SG002 IgA levels in bile juice samples were not significantly different from the negative control using peptide- and OMP-ELISAs (Figures 5A–D).

The inoculation of SG0197 in 8-w-o male brown layer chickens did not cause mortality in either the SG 9R vaccine or no vaccine (CONT) group at 2 wpi. The SG 9R vaccine and no vaccine groups did not display different OD values for the peptide-ELISA (Figures 5E–G). However, the SG 9R vaccine group showed significantly higher OD values than the no vaccine group using the OMP-ELISA (*P* < 0.05) and O-Ag-ELISA (Figures 5H,I).

### Humoral Immunity Against SG in the Field

**Figure 6:**
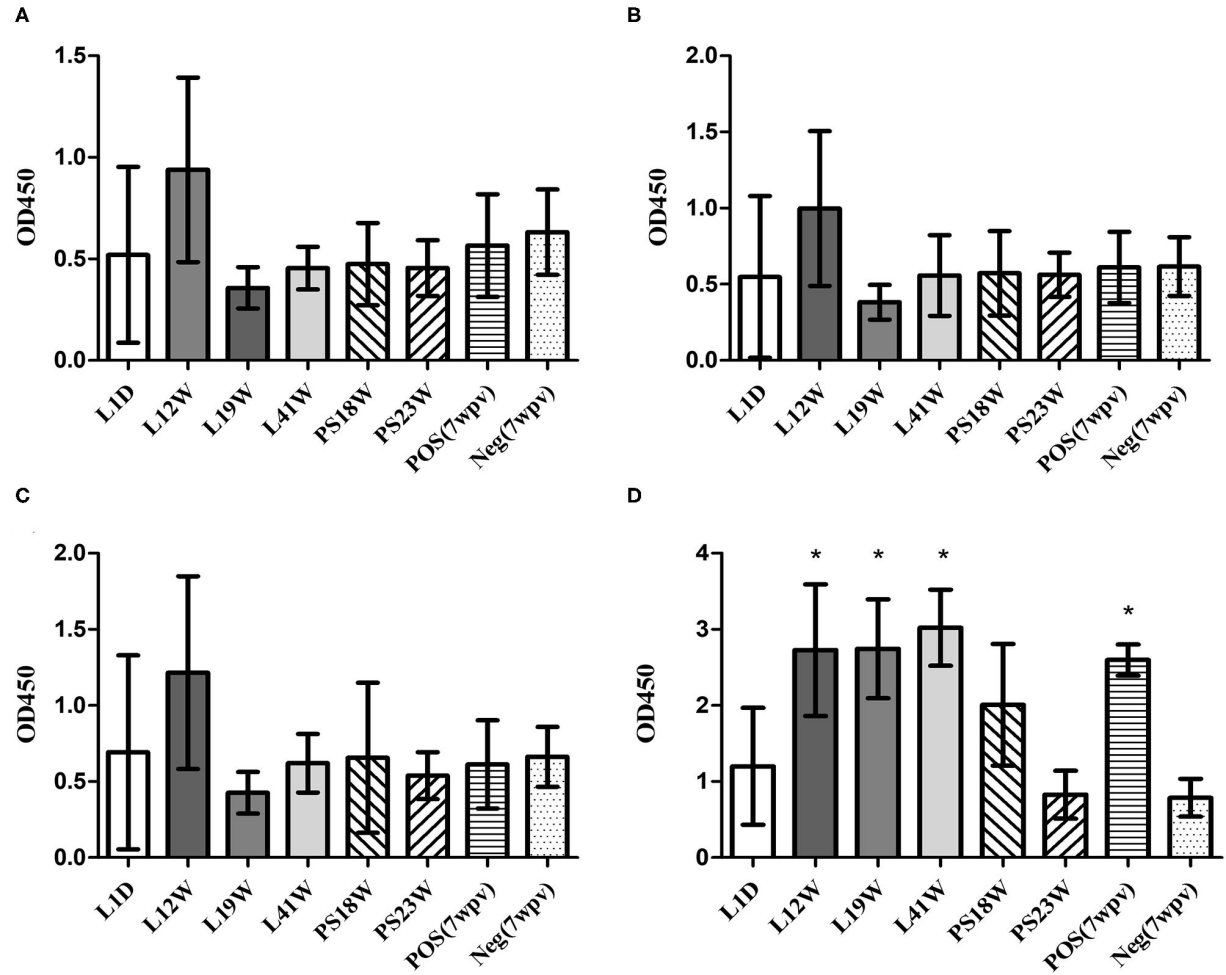
Humoral immunity against SG in the field (mean with SD): OmpA-N-L3 **(A)**, OmpA-N-L4 **(B)**, OmpX-L2 **(C)**, and OMP-ELISA **(D)**. The field serum samples from the L1D (1 d-o), L12W (12 w-o, vaccination at 10 weeks of age), L19W (19 w-o, vaccination at 8 and 15 weeks of age), and L41W (41 w-o, vaccination at 7 and 16 weeks of age) groups were collected from laying hens, and the PS18W (18 w-o) and PS23W (23 w-o) samples were collected from parent stocks. *Indicates a significant difference compared with Neg. [*P*-values - **(D)** L12W (0.002), L19W (0.002), L41W (0.000), and POS (0.028)].

When the field serum samples were tested using peptide-ELISAs, L12W showed higher OD values than the other samples, although the differences were not significant. The OMP-ELISA revealed significantly higher OD values in the L12W, L19W, and L41W groups than in the negative control (Figure 6D).

## Discussion

Fowl typhoid vaccines are categorized into live attenuated and inactivated vaccines, and live attenuated vaccine strains are subdivided into rough and smooth strains (15–17). As O-Ag of LPS hides OMPs and induces strong activation of specific B cells, the immunogenicity of OMPs of smooth strains may be less than rough strains (18). Our western blotting results from 2D-gel electrophoresis with whole bacterial lysates revealed predominant humoral immunity to O-Ag, and the results of the peptide- and OMP-ELISAs of SG002 and SR2-N6 supported the hypothesis that OMPs of the rough strain are more immunogenic than OMPs of the smooth strain (Figures 1, 2). The increased immunogenicity of OMPs may be due to unrestricted exposure to B cells without shielding by O-Ag and the absence of a competing strong immunogen monopolizing most of the resources of humoral immunity. Considering the already improved protective efficacy of OMP vaccines and antigenic conservation among Gram-negative bacteria, the potential value of rough strains to become universal vaccines needs to be demonstrated in future studies (19).

SG 9R has been used worldwide due to its better protection efficacy, but the humoral immune responses to live, killed and a mixture of live, and killed SG 9R have never been compared. The significantly lower immunogenicity of PS SG 9R than SG 9R was unexpected because a killed rough strain of *Salmonella* serovar Typhimurium generated a higher antibody titer than the live rough strain (18). We killed the bacteria at 65°C for 2 h, while the authors of the previous used 100°C for 30 min and subsequent treatment with 1% human serum albumin and 0.16% formaldehyde. Therefore, the additional treatment with albumin and formaldehyde may have resulted in different results. Interestingly, a synergistic effect of PS SG 9R and live SG 9R was apparent and may reflect cooperative stimulation of humoral immunity by dead and live bacteria. Therefore, this new formulation without the use of the carcinogen formaldehyde may be useful to improve the protective efficacy of conventional live vaccines against infection with virulence variants recently detected in the field (20).

The oil adjuvant significantly increased the immunogenicity of OE SG 9R compared with PS SG 9R and generated similar serum antibody titers to SG 9R. Because of its potential pathogenicity, SG 9R vaccination of chickens aged <6 w-o is not recommended (8, 21, 22). Because the highest susceptibility and likelihood of infection are observed during the prevaccination ages (1 d-o to 6 w-o or age before vaccination), clinical measures, including early inoculation with adjuvanted killed vaccines, can be considered to protect chicks from vertical and/or horizontal transmission of SG.

As invasive *Salmonella enterica* serovars penetrate the cell as an intracellular pathogen, researchers assumed that live bacteria might not produce sufficiently high titers of specific antibodies in the bloodstream. SG did not stimulate the initial immune response via proinflammatory cytokines or chemokines due to the absence of flagella, and the ability of SG to evade the immune system was very remarkable, even when the systemic infection had progressed (23, 24). Consistent with previous reports, infection with a field strain might delay humoral immunity by ~2 weeks compared with SG 9R inoculation (Figure 4) and might result in insignificant mucosal antibody levels compared with the negative control (Figure 5). However, we should consider their different routes of infections. The observation that surviving chickens mounted antibodies against OmpX peptides and OMPs may support the importance of humoral immunity in survival. Additionally, a significant increase in the IgA titer in the bile juice of SG 9R-vaccinated chickens may support the importance of humoral immunity induced by SG 9R vaccination (Figure 5). Because most commercial layer farms inoculate animals with SG 9R vaccines, testing IgA levels in bile juice, intestinal washes and feces may be useful for the differential diagnosis of FT and an estimation of the risk of SG exposure.

Considering temporary increases in OD values obtained from peptide-ELISAs during the first 2 weeks after SG vaccine inoculations, the higher OD value of L12W may be related to the SG 9R vaccination at 10 weeks of age (Figure 6). Thus, the peptide-ELISAs were able to detect specific antibodies induced by recent SG 9R vaccination under both experimental and field conditions. However, the OMP-ELISA revealed significantly higher antibody levels in vaccinated flocks than in unvaccinated flocks. All field samples were tested using the O-Ag ELISA, and no positive sample indicating a field strain or SE infection was examined (data not shown). Therefore, these assays may be useful to verify the efficacy of the inoculated vaccine and monitor unlawful vaccination with parent stocks in the field in combination with the O-Ag ELISA.

The immunogenic OMPs of *Salmonella enterica* have been reported, and OmpA and OmpX are known to be protective antigens (25–27). Although we selected OmpA and OmpX due to their immunodominance, the rapid but short-lived antibody responses induced by these antigens were unexpected and have not been reported. Additionally, our study is the first to investigate the kinetics of the production of specific antibodies against linear epitopes of OmpA and OmpX compared with OMPs. In summary, with the gradual increase in the titers of antibodies against OMPs over time, there may be other OMPs inducing long-lasting antibody responses. Although the immune dominance of the C-terminus of OmpA was reported previously, we could not find any difference between the results of C- and N-terminal peptide-ELISAs (Supplementary Figure 1) (28).

In conclusion, rough strains are better than smooth strains in terms of the immunogenicity of OMPs, and a mixture of a live and killed rough vaccine strains may potentiate the efficacy of the conventional live vaccine. The evasion of humoral immunity by the field strain was demonstrated again, but SG 9R may be useful to prime mucosal immunity against infection with a field strain. Additionally, combined serological tests with peptide, OMP, and O-Ag ELISAs may be useful for the differential diagnosis of FT in the field.

## Data Availability Statement

The original contributions presented in the study are included in the article/Supplementary Materials, further inquiries can be directed to the corresponding author/s.

## Ethics Statement

The animal study was reviewed and approved by Institutional Animal Care and Use Committee of BioPOA Co.

## Author Contributions

N-HK, H-JK, and K-SC substantially contributed to conceptualization, data curation, and analysis of the study. H-JK supervised all surveillance components. E-JH and D-SK contributed to analysis of data. N-HK prepared the initial draft, figures, and tables. N-HK and H-JK contributed to the writing and editing of the manuscript. All authors contributed to the article and approved the submitted version.

## Conflict of Interest

The authors declare that the research was conducted in the absence of any commercial or financial relationships that could be construed as a potential conflict of interest.
