# Supplementary material for: Comparison of Humoral Immune Responses to Different Forms of Salmonella enterica Serovar Gallinarum Biovar Gallinarum
Source: Front Vet Sci. 2020 Nov 6;7:598610. doi: 10.3389/fvets.2020.598610 (PMC7677237; doi:10.3389/fvets.2020.598610)
Supplement: Supplementary file 1 [file Table_1.DOCX]

**Supplementary Table 1. 2DE western blotting and LC-MS / MS analysis were performed using whole bacteria as antigen.**

| **Band label** | **NCBI BLAST** | **Protein name** | **Score** | **Mass** |
| --- | --- | --- | --- | --- |
| **A** | gi\|1028875858 | translation elongation factor G [Salmonella enterica] | 568 | 77788 |
| **B** | gi\|446651785 | molecular chaperone GroEL [Salmonella enterica] | 1342 | 57382 |
| **C** | gi\|513039272 | phosphoglycerate kinase [Salmonella enterica] | 601 | 41310 |
| **D** | gi\|823641791 | elongation factor Tu [Escherichia coli] | 311 | 43486 |
| **E** | gi\|446079918 | electron transfer flavoprotein subunit beta [Salmonella enterica] | 419 | 33608 |
| **F** | gi\|972719165 | elongation factor TU [Erwinia gerundensis] | 222 | 45118 |
| **G** | gi\|321222631 | 3~(2~),5~-bisphosphate nucleotidase [Salmonella enterica subsp. enterica serovar Typhimurium str. TN061786] | 185 | 25890 |
| **H** | gi\|353561131 | Endoribonuclease L-PSP [Salmonella enterica subsp. enterica serovar Gaminara str. A4-567] | 90 | 14141 |
| **I** | gi\|518056966 | MULTISPECIES: cold-shock protein [Firmicutes] | 83 | 7257 |
