# Supplementary figures and images for: Comparison of Humoral Immune Responses to Different Forms of Salmonella enterica Serovar Gallinarum Biovar Gallinarum
Source: Front Vet Sci. 2020 Nov 6;7:598610. doi: 10.3389/fvets.2020.598610 (PMC7677237; doi:10.3389/fvets.2020.598610)

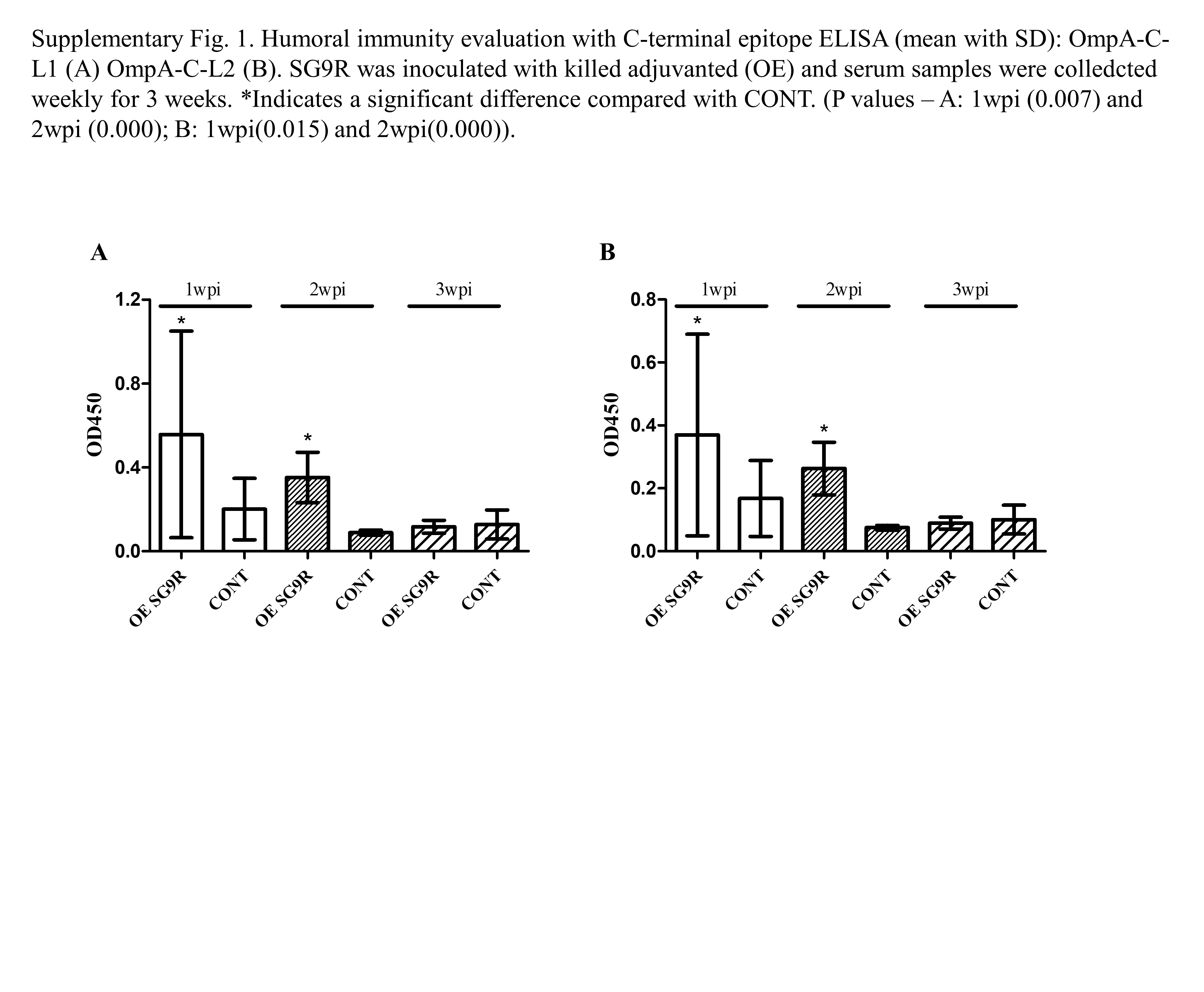

Supplement: Supplementary file 2 [file Image_1.TIF]
